# Lifestyle interventions in cardiometabolic HFpEF: dietary and exercise modalities

**DOI:** 10.1007/s10741-024-10439-1

**Published:** 2024-09-16

**Authors:** Antonio Vacca, Rongling Wang, Natasha Nambiar, Federico Capone, Catherine Farrelly, Ahmed Mostafa, Leonardo A. Sechi, Gabriele G. Schiattarella

**Affiliations:** 1 Deutsches Herzzentrum Der Charité (DHZC), Charité-Universitätsmedizin Berlin, Berlin, Germany; 2 DZHK (German Centre for Cardiovascular Research), Partner Site Berlin, Berlin, Germany; 3 Translational Approaches in Heart Failure and Cardiometabolic Disease, Max Delbrück Center for Molecular Medicine in the Helmholtz Association (MDC), Berlin, Germany; 4 Division of Internal Medicine, Department of Medicine-DIMED, University of Padua, Padua, Italy; 5 Clinica Medica, Department of Medicine, University of Udine, Udine, Italy; 6 Division of Cardiology, Department of Advanced Biomedical Sciences, Federico II University, Naples, Italy

**Keywords:** HFpEF, Obesity, Diet, Exercise

## Abstract

Heart failure with preserved ejection fraction (HFpEF) is rapidly growing as the most common form of heart failure. Among HFpEF phenotypes, the cardiometabolic/obese HFpEF — HFpEF driven by cardiometabolic alterations — emerges as one of the most prevalent forms of this syndrome and the one on which recent therapeutic success have been made. Indeed, pharmacological approaches with sodium-glucose cotransporter type 2 inhibitors (SGLT2i) and glucagon-like peptide-1 receptor agonists (GLP-1RA) have proved to be effective due to metabolic protective effects. Similarly, lifestyle changes, including diet and exercise are crucial in HFpEF management. Increasing evidence supports the important role of diet and physical activity in the pathogenesis, prognosis, and potential reversal of HFpEF. Metabolic derangements and systemic inflammation are key features of HFpEF and represent the main targets of lifestyle interventions. However, the underlying mechanisms of the beneficial effects of these interventions in HFpEF are incompletely understood. Hence, there is an unmet need of tailored lifestyle intervention modalities for patients with HFpEF. Here we present the current available evidence on lifestyle interventions in HFpEF management and therapeutics, discussing their modalities and potential mechanisms.

## Introduction

Heart Failure with preserved ejection fraction (HFpEF) currently represents the most common form of heart failure (HF) [1], and its prevalence is increasing by 10% per decade relative to HF with reduced ejection fraction (HFrEF) [2]. This gap is expected to increase further in the coming years as a result of the cardiovascular aging of the population and the increasing prevalence of HFpEF-predisposing conditions, such as hypertension, obesity, metabolic syndrome (MetS), and diabetes in particular [3, 4]. Although HFpEF presents with similar symptoms as in HFrEF, it shows different pathophysiological mechanisms, with the transition from HFpEF to HFrEF being rare [3, 5]. HFrEF cornerstone neurohormonal therapies have failed to improve outcomes in HFpEF, shifting the therapeutic target in HFpEF towards metabolic-based pharmacological strategies [6]. Indeed, only novel pharmacological approaches such as sodium-glucose cotransporter type 2 inhibitors (SGLT2i) and glucagon-like peptide-1 receptor agonists (GLP-1RA) have revealed favorable impacts on clinical outcomes in HFpEF, improving quality of life of patients due to their metabolic protective effects [7, 8].

HFpEF presents a large phenotypical heterogeneity coupled with a high comorbidity burden and a complex multiorgan systemic pathophysiology [9]. Among various HFpEF phenotypes, the cardiometabolic/obese HFpEF — elicited by metabolic alterations — represents the most prevalent form of this syndrome [10]. According to the World Obesity Atlas 2023 report, 38% of the population worldwide is currently either overweight or obese, and by 2035, the global overweight and obesity prevalence is expected to reach 51% [11]. A body mass index (BMI) of > 25 kg/m^2^ is associated with a greater risk of HFpEF than HFrEF [12] and more than 80% of patients with HFpEF are overweight or obese [13]. Obesity contributes to risk factors for MetS, a condition characterized by the coexistence of visceral adiposity, dyslipidemia, type 2 diabetes, and hypertension strongly predicting HFpEF [14]. The increasing prevalence of diabetes is also reported worldwide by epidemiological data, raising from 30 to 400 million people since 1985 [15]. Western diet (WD), composed of high saturated fat and sugar [16] and associated with a Western lifestyle of sedentary behavior in the form of prolonged sitting during work and transportation [17], is an important modifiable risk factor for cardiometabolic HFpEF. Saturated fats and refined carbohydrates produce a high caloric influx into adipose tissue and often exceed the storage capacity of adipocytes. This causes increased serum lipids, enhanced lipid uptake by non-adipose tissues, and ectopic lipid accumulation [18].

The American Heart Association created “Life’s simple 7” measures to achieve ideal cardiovascular health including (1) quitting smoking, (2) eating healthy, (3) being active, (4) losing weight, (5) managing blood pressure (BP), and (6) controlling of cholesterol and (7) plasma glucose levels [19]. Diet and lifestyle changes play a pivotal role in the prevention and treatment of cardiovascular disease and beneficial effects based on AHA measures are well documented in HF [20]. Complying with these measures seems to be particularly important for HFpEF [21]. Indeed, dietary habits have been involved in the pathogenesis [22–24], prognosis [25–27], and potential reversal of HFpEF [28]. Similarly, in HFpEF, exercise training shows beneficial effects on diastolic disfunction, enhances skeletal muscle structure and function, and reduces adiposity and inflammation [29–32]. However, the specific impact of different types of lifestyle intervention on mechanisms of HFpEF remains largely unknown.

## Metabolic derangements and systemic inflammation in cardiometabolic HFpEF

**Fig. 1:**
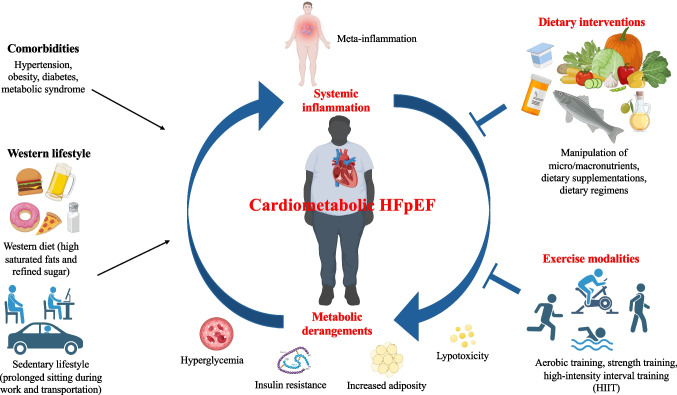
Scheme depicting predisposing conditions of cardiometabolic stress driving HFpEF as well as main pathophysiological features of HFpEF and potential lifestyle interventions. Created with BioRender.com licensed to G.G.S

Metabolic derangements and systemic inflammation are reported as the main pathophysiological features of HFpEF (Fig. 1) [18]. The presence of insulin resistance (IR) and oxidative stress is well documented in HFpEF, resulting as the common hallmarks of cardiometabolic comorbidities [15, 33, 34]. IR worsens glucose uptake and utilization in cardiomyocyte and triggers cardiac metabolic remodeling, shifting from glucose oxidation to fatty acids oxidation (FAO) via the Randle cycle [35]. Altered cardiac substrate utilization in HFpEF is another key aspect of HFpEF pathophysiology triggering metabolic remodeling. Clinical [36, 37] and pre-clinical [38] studies suggested suppressed fatty acids (FAs) metabolism in HFpEF hearts, using indirect measurements of cardiac energy metabolism. However, direct flux measurements revealed an altered metabolic profile towards a switch in substrate utilization from glucose oxidation to FAO [39]. These findings are in line with previous results obtained in obesity and diabetes, showing up-regulated FAO [39–41] accompanied by decreased glucose oxidation [42–44]. Increased lipolysis in adipose tissue due to IR [45] and excessive reliance on free FAs [18] are linked to up-regulated uptake of FAs in cardiomyocytes [39]. The resulting lipids overload leads to the accumulation of lipotoxic intermediates — as diacylglycerols (DAGs), ceramides, and triglycerides (TGs) [35] — oxidative stress [18], and altered ATP production [46].

A healthy adult heart requires around 6 kg of ATP per day, representing a highly energy-demand organ [47]. Diastole — in which ATP is used to break actomyosin  cross-links allowing cardiac relaxation — represents the most energetically demanding phase of cardiac cycle [48]. Most of ATP heart sources rely on free fatty acids (FFAs) oxidation (~ 70%), with glucose, ketone bodies (KBs), and amino acids playing a complementary role as alternative substrates [35]. Healthy cardiac tissue is metabolically flexible, adapting its substrate usage based on nutrient availability, local and systemic conditions, allowing ATP generation to continue in fed, fasting, and high-demand states [49]. Conversely, a failing heart is typically characterized by a loss of metabolic flexibility [49] and fails to respond to dynamic changes in energy demand. Indeed, patients with HFpEF show a 20–27% reduction in phosphocreatine (PCr)/adenosine triphosphate (ATP) ratio, which represents an index of the energetic state of the heart and reflects the balance of energy consumption and energy supply in the heart [50–52]. ATP provides a direct energy source for cellular reactions, while PCr acts as an energy storage and transport compound via the “creatine kinase-PCr energy shuttle” [53]. PCr buffers ATP in cardiomyocytes during high demand conditions. A low ratio between these high-energy phosphate compounds in human hearts, as non-invasively assessed with ^31^Phosphorus magnetic resonance spectroscopy (^31^P-MRS), suggests compromised mitochondrial function [54]. Previous clinical studies demonstrated that this ratio is reduced in failing human myocardium [53]. In obesity and diabetes loss of metabolic flexibility is associated with impaired glucose oxidation and concomitant cardiac hypertrophy and dysfunction [55]. Thus, strategies aiming to restore the resilience between energy substrates are warranted to maintain the ATP production in HFpEF [56].

A systemic low-grade inflammation stemming from comorbidities-driven metabolic derangements (i.e., meta-inflammation) represents the other key feature of HFpEF [57], implying an increased burden of oxidative and nitrosative stress [58, 59]. Metabolic derangements, such as hyperglycemia and increased adiposity, promote the release of cytokines and pro-inflammatory adipokines, triggering systemic inflammation and immune alterations [18, 60, 61]. Moreover, evidence in hypertensive patients reports an association between hyperglycaemia and increased risk of diastolic dysfunction even in the absence of diabetes [62]. Adipocyte-derived saturated FAs (SFAs) activate toll-like receptor 4 (TLR4) in macrophages, causing the release of tumor necrosis factor-alpha (TNF-α) and interleukin-6 (IL-6) [18]. The latter affects directly cardiomyocytes, stimulating mitogen-activated protein kinases (MAPKs) and nuclear factor kappa-light-chain-enhancer of activated B (NFkB) signaling, inhibiting Akt, and promoting diastolic dysfunction [63, 64]. Systemic meta-inflammation, together with the paracrine effects of epicardial tissue, elicits HFpEF cardiac remodeling through increasing cardiomyocyte hypertrophy and myocardial fibrosis [18].

Moderate weight loss of 5–10 kg through dietary and exercise interventions results in a clinically meaningful reduction of cardiometabolic risk [65]. Interestingly, weight loss leads to lower myocardial oxygen consumption and decreased myocardial FAO [66], increasing myocardial glycolysis, myocardial glucose oxidation [67], and PCr/ATP in obese patients [68]. In addition, weight loss decreases circulating lipids, improves IR and inflammation [69, 70] and reduces systolic BP by at least 1 mmHg per kg of weight loss [71].

## Dietary management of HFpEF

Dietary management of HFpEF provides benefits to the cardiovascular and muscle-skeletal system as a whole [20]. Importantly, most of the evidence to date were collected in HF mixed populations, with a limited number of studies focusing on HFpEF subjects (Table 1).

**Table 1:** Evidence of nutritional interventions in HFpEF and HF mixed populations: manipulation of micro/macronutrients or modulation of specific clinical traits, dietary supplementations, and dietary regimen interventions

Category	Interventions	Evidence		
**Manipulation of micro/macronutrients or modulation of specific clinical traits**	**Salt intake**	No clear evidence of benefits after 30 days of sodium (0.8 g/d) and fluid (800 mL/d) restriction Association between overstrict dietary salt restriction and worse prognosis over a period of 3 years		Machado d’Almeida et al. [17] Li J et al. [77]
	**UFAs**	Improvement in CRF and clinical outcomes after 12 weeks of increased UFAs consumption. Recommended daily amount (or more, without upper limit for consumption) of UFA-rich foods: extra-virgin olive oil (54 g), canola oil (54 g), unsalted or lightly salted mixed dry tree nuts (walnuts, hazelnuts, almonds, pecans), and peanuts (28 g), without providing recommendations on caloric intake. In patients who could not consume the recommended foods unsalted mixed seeds (28 g), Hass avocado (50 g), and fatty fish (salmon, tuna, trout, mackerel, sardines) (170 g)		Carbone et al. [85]
	**Carbohydrates intake**	Improvement in oxygen saturation in HF mixed population after 2 months of LC diet (< 130 g/d of carbohydrates) Benefits in weight loss in patients with DMCM after 16 weeks of LC diet (< 130 g/d of carbohydrates) LC diet may improve IR and metabolic function in DMCM		González-Islas et al. [89] Kleissl-Muir et al. [87] Kleissl-Muir et al. [86]
	**CR and obesity**	Improvement in VO_2_ peak. Combined CR (approximately 350 kcal/d) and exercise for 20 weeks show additional effects Improvements in cardiac function after CR diet to achieve a weight loss of > 5 kg within 6 months Calorie-restricted high-protein diet for 3 months (30% protein, 40% carbohydrates, and 30% fat) reduces cardiometabolic risk in a mixed HF population		Cocco et al. [95] Kitzman et al. [94] Evangelista et al. [101]
**Dietary supplementations**	**IN**	BP lowering effect up to 4 weeks of supplementation (6.4 mmol/d of oral nitrate) No significant benefits in improving exercise capacity in HFpEF after 12 weeks of supplementation (40 mg, three times daily)	Lv et al. [107] Borlaug et al. [109]	
	**CoQ**_**10**_	Some clinical trials showed improvements in symptoms and adverse cardiovascular events, while others found no significant effects	Mortensen et al. [113] Sobirin et al. [114] Pierce et al. [115] Samuel et al. [116]	
	**L-carnitine**	Benefits in weight loss after 1 year of supplementation (300 mg/d)	Kinugasa et al. [122]	
	**Vitamin D3**	No clear evidence of benefits in a mixed HF population after 6 months of supplementation (50,000 IU of vitamin D3 daily and calcium citrate 400 mg twice daily)	Boxer et al. [130]	
	**UFAs**	Lower mortality and hospitalization (ω-3 PUFAs supplementation) in a mixed HF population followed up for a median of 3.9 years Improvement in related-HF cognitive depressive symptoms and physical function after 12 weeks of supplementation [EPA + DHA (2:1 ratio, four capsules of 400/200 mg EPA/DHA out of the total of 500 mg of ω-3 UFAs per capsule daily) and EPA alone (four capsules of almost pure EPA 500 mg per capsule daily)] in a mixed HF population	Tavazzi et al. [137] Jiang et al. [138]	
	**Proteins**	Improved physical and cardiovascular function after 12 weeks of protein supplementation (1.2 g/kg bodyweight per day) combined with low-intensity exercise	Azhar et al. [139]	
	**BCAAs**	No clear evidence of benefits in a mixed HF population after 3 months of supplementation (10 g/d)	Pineda-Juárez et al. [140]	
**Dietary regimens**	**DASH/SDR diet**	Benefits in BP, diastolic LV relaxation, chamber stiffness, and ventricular-arterial coupling in a mixed HF population after 21 days of DASH/SDR diet	Hummel et al. (2012) Hummel et al. [144]	
	**LED**	Reverse of cardiovascular remodeling in diabetic obese patients after 16 weeks of a VLCD Weight loss and improvement of diabetes-related cardiometabolic risk after 12–20 weeks of low-energy MRP Improved myocardial steatosis and diastolic filling in type 2 diabetes after 12 weeks of low-energy MRP	Hammer et al. [145] Lean et al. [147] Gulsin et al. [146]	
	**KD**	Benefits in weight loss after 24 weeks of KD Cautious application required: detrimental effects on cardiovascular health, contributing to cardiac lipotoxicity and adversely modifying cardiac muscle energy metabolism	Nordmann et al. [148] Lopaschuk et al. [151]	
	**MedDiet**	Reduced HF hospitalizations after 1 year of adherence to MedDiet Benefits in systemic inflammation in patients with HF and metabolic syndrome after 1 year of adherence to MedDiet	Casas et al. [156] Fitó et al. []157 Miró et al. [155]	

*BCAA* branched chain amino acids, *BP* blood pressure, *CR* caloric restriction, *CRF* cardiorespiratory fitness, *CoQ*_*10*_ coenzyme Q_10_, *DASH/SDR diet* dietary approach to stop hypertension/sodium restricted diet, *DHA* docosahexaenoic acid, *DMCM* diabetic cardiomyopathy, *EPA* eicosapentaenoic acid, *HF* heart failure, *IN* inorganic nitrate, *KD* ketogenic diet, *LC* low carbohydrate, *LED* low energy diet, *LV* left ventricle, *MedDiet* Mediterranean diet, *MRP* meal replacement plan, *UFAs* unsaturated fatty acids, *VLCD* very-low-calorie diet

### Manipulation of micro/macronutrients or modulation of specific clinical traits

Manipulation of single micro/macronutrients or modulation of a specific clinical trait has been adopted as a potential dietary strategy for patients with HFpEF.

Management of salt intake has been associated with significant amelioration in quality of life (QoL) and outcomes in HF subjects, reducing congestion and edema [72–75]. However, the effects of sodium restriction in HFpEF remain controversial. Aggressive sodium and fluid restriction in 53 decompensated HFpEF patients showed no neurohormonal benefits [76]. In addition, an observational study [77] analyzing data from the TOPCAT trial [78] found an association between overstrict dietary salt restriction and worse prognosis in HFpEF patients.

Unsaturated fatty acids (UFAs) comprise monounsaturated fatty acids (MUFAs) and polyunsaturated fatty acids (PUFAs) and are associated with favorable cardiovascular outcomes in obese and hypertensive patients [27, 79]. Foods rich in MUFAs are olive oil, avocados, nuts, and seeds, while sources of PUFAs are fatty fish, flaxseeds, chia seeds, walnuts, sunflower, and corn oil [79, 80]. Most MUFAs and ω-3 PUFAs, such as alpha-linolenic acid (ALA), eicosapentaenoic acid (EPA), and docosahexaenoic acid (DHA) show protective effects for metabolic and physiological processes, as well as inflammatory response [80]. The beneficial effect of UFAs on improving insulin sensitivity has been reported in vitro [81, 82] and in vivo studies [83, 84]. Studies on evaluating the effects of UFAs for HFpEF patients are extremely limited. Only one completed trial (NCT03310099) reported that an UFA-rich foods diet consumption for 84 days in 9 obese symptomatic HFpEF patients improved cardiorespiratory fitness (CRF) and clinical outcomes [85] and one on-going trial [the UFA-Preserved 2 (NCT03966755)] is set to follow up on this. Further studies are needed to fully understand the role of UFAs in the management of HFpEF.

Carbohydrate manipulation may represent another dietary strategy in HFpEF. Significant weight reduction following a low carbohydrate diet for 16 weeks (ACTRN12620001278921) is reported in patients with diabetic cardiomyopathy (DMCM) [86]. A low carbohydrate diet, which falls below 130 g of carbohydrates per day, may improve systemic IR, whole-body metabolism, and tissue functions [87]. Moreover, it shows favorable effects on low-grade inflammation in patients with type 2 diabetes (T2D) [88]. A low carbohydrate diet for 2 months was found to improve oxygen saturation in HF [89], but the clinical relevance could not be established [90].

A strategy for obesity management is calorie restriction (CR). Indeed, CR — i.e., reduction of caloric intake by 30–40% — shows positive effects on cell metabolism, resulting in weight loss and reducing systemic inflammation and oxidative stress [91]. Moreover, CR improves metabolic parameters, such as insulin sensitivity and lipid metabolism [92, 93]. CR or interventions aiming to rescheduling the time of feeding during the day [intermittent fasting and time restricting eating (TRE)] demonstrated to reduce cardiovascular events in obesity, diabetes, and metabolic syndrome [91]. A significant improvement in VO_2_ peak was shown in 100 obese patients with HFpEF treated with CR and aerobic exercise training for 20 weeks (NCT00959660), suggesting an additive effect of both interventions in obese HFpEF [94]. In addition, a 6-month CR diet program in 38 obese hypertensive HFpEF patients followed by > 5 kg weight reduction, led to reduction in NT-proBNP circulating levels. This was followed by an improvement in diastolic function and 6 minute walk distance (6MWD) [95]. CR inhibits the IGF-1/insulin pathway and improves protein quality control in skeletal muscle [96]. CR may reverse mitochondrial dysfunction in aging muscle stem cells (MuSCs) restoring myofiber growth and intrinsic muscle function, showing beneficial effects on muscle oxygen supply, exercise capacity, and QoL of HFpEF patients [97]. In support of this, intermittent fasting, reached by limiting caloric intake to 8 hours during the day-time, reduced cardiovascular risk in resistance-trained men [98]. In addition, a program of 10 hours-TRE for 12 weeks reduced BP and LDL cholesterol levels in patients with MetS [99]. Intermittent fasting and TRE induced a shift from fat to ketone metabolism and modulation of cellular adaptive responses, such as autophagy [91, 100]. A calorie-restricted high-protein diet for 3 months (30% protein, 40% carbohydrates, and 30% fat) in a HF population, including a 43.3% of HFpEF subjects [90], reduced cardiometabolic risk with significant improvements in BP in comparison to a standard-content protein diet (15% protein, 55% carbohydrates, and 30% fat) [101]. Thus, CR shows significant cardiometabolic effects, such as improving cardiorespiratory fitness (CRF), reducing body weight, ameliorating insulin sensitivity and glucose metabolism, improving lipid profile, and reducing systemic inflammation. These effects make CR potentially clinically relevant in the treatment of patients with HFpEF.

Supplementation of several synthetic and natural compounds — known as calorie restriction mimetics (CRMs) — may represent a valid alternative to CR, mimicking its physiological and molecular effects [91]. Examples of CMRs are represented by spermidine, resveratrol, curcumin, and epigallocatechin-3-gallate, which have shown promising results in mouse models [91]. In particular, spermidine — a naturally occurring polyamine found in soybeans, mature cheese, mushrooms, and broccoli — promotes cardioprotective autophagy [102] and attenuates cardiac senescence due to prevention of oxidative stress and improvement in mitochondrial function in preclinical HFpEF [103, 104]. In addition, the anti-inflammatory properties of spermidine are reported through inducing anti-inflammatory (M2) macrophage expression and decreasing TNF-α levels [105, 106]. Clinical implications of spermidine supplementation in HFpEF and cardiometabolic diseases are currently still unclear.

### Dietary supplementations

Dietary supplementation studies tested the effects of dietary micro/macronutrients in HFpEF in the form of tablet, capsule, liquid, or powder.

Inorganic nitrate/nitrite (IN) supplementation in HFpEF showed BP lowering effect, especially during exercise [107]. Moreover, the anti-inflammatory effects of IN supplementation in atherosclerosis and systemic inflammation have been reported [108]. However, a recent clinical trial in 92 patients with HFpEF (NCT02713126) demonstrated that IN supplementation (40 mg, three times daily) for 12 weeks did not provide additional benefits from exercise training [109]. A meta-analysis of 8 randomized controlled trials (RCTs) confirmed the absence of benefits of IN supplementation in improving exercise capacity in HFpEF [107].

The REDUCE-IT trial revealed the potential therapeutic benefit of icosapent ethyl supplementation in reducing cardiovascular risk by targeting inflammation in patients with HF (NCT01492361). Icosapent ethyl showed anti-inflammatory properties by reducing the level of high-sensitivity C-reactive protein (hs-CRP) as an inflammatory biomarker, alongside its lowering effects on triglyceride levels from baseline to 2 years compared to placebo [110]. Further studies are needed to confirm the veracity of these effects in the context of systemic inflammation in patients with HFpEF.

Coenzyme Q_10_ (CoQ_10_) supplementation in T2D *db*/*db* mouse models revealed attenuation of diastolic dysfunction and cardiac remodeling [111]. Supplementation of CoQ_10_ is also associated with a rise in adiponectin levels, which in turn leads to a decrease in inflammatory response mediated by TNF-α [112]. The Q-SYMBIO trial [113] reported that a long-term CoQ_10_ supplementation reduced major adverse cardiovascular events and improved symptoms in patients with chronic HF, including 7% HFpEF [90]. In addition, a short-term CoQ_10_ supplementation (30 days) in 30 HFpEF patients led to statistically significant within-group changes in diastolic function, despite these were not significantly different from the control [114]. Ubiquinol — the active form of CoQ_10_ — and D-ribose showed a positive impact on HFpEF symptoms in a RCT study (NCT03133793) [115]. However, a RCT in elderly HFpEF patients (NCT02779634) reported no effects of CoQ_10_ supplementation (100 mg, three times daily) on diastolic function [116].

L-carnitine is an amino acid derivative that plays a critical role in lipid metabolism through transporting long-chain FAs to mitochondria for oxidation [117]. Decreased L-carnitine content has been reported in the failing heart [118]. L-carnitine may prevent myocardial fibrosis and HFpEF, through enhanced production of prostacyclin [119], and has been shown to promote weight loss, improve IR, and reduce appetite and food intake through a direct effect on the hypothalamus in obese adults [120]. In addition, administration of L-carnitine in animal with myocardial infarction shows effects in reducing oxidative stress and enhancing antioxidant enzyme activity through the inhibition of TNF-α and IL-1β [121]. Eighteen patients with HFpEF, presenting reduced L-carnitine at the baseline level, were supplemented with L-carnitine (300 mg daily) for 1 year (UMIN000011905) [122]. The study reported significant weight loss but no improvements in left ventricular (LV) diastolic function.

Vitamin D (VD) deficiency is associated with reduced functional capacity in patients with diastolic dysfunction or HFpEF [123]. Low VD levels are also associated with impaired glucose tolerance in nondiabetic hypertensive patients and may contribute to organ damage [124]. Serum 25-hydroxyvitamin D [25(OH)D] levels < 50 nmol/L have been associated with increased LV mass and LV hypertrophy in hypertensive patients [125]. VD appears to have cardiovascular protective effects by modulating inflammatory cytokines, reducing oxidative stress, and regulating the systemic renin–angiotensin–aldosterone system [126, 127]. VD supplementation improves glycaemic homeostasis and insulin sensitivity among adults at risk for T2D [128] and showed anti-inflammatory properties in a population of healthy Saudi males [129]. These effects point to potential positive effects on cardiometabolic health in patients with HFpEF. However, a 6-month VD supplementation (50,000 IU of vitamin D3 daily and calcium citrate 400 mg twice daily) in a mixed HF population (NCT01125436) showed no beneficial effects on aerobic capacity and physical performances [130].

Subtle differences in regulation of cortisol levels in hypertensive patients are associated with impaired glucose tolerance and IR [131], and minimal excess of cortisol in hypertensive patients contributes independently to LV hypertrophy and concentric remodeling, potentially contributing to LV diastolic dysfunction and HFpEF [132]. Supplementation of ω-3 PUFAs showed an association with lower cortisol levels and inflammation [133] and prevented fibrosis and diastolic dysfunction in transverse aortic constriction (TAC) animal models with pressure overload-induced cardiac hypertrophy, by activation of the cyclic guanosine monophosphate (GMP)/protein kinase G pathway in cardiac fibroblasts [134]. These findings suggest the clinical potential of ω-3 PUFAs supplementation. The MESA study [135] found an association between higher plasma EPA and lower risk of HF, including HFrEF and HFpEF. In addition, a retrospective study on 140 hospitalized decompensated HFpEF patients indicated that low DHA plasma levels were associated with an increase in all-cause death, suggesting a potential role of DHA for diagnosis and therapies in such patients [136]. The GISSI-HF trial in a mixed HF population (HFrEF and HFpEF), including 634 patients with HFpEF [90], revealed beneficial effects of treatment with ω-3 PUFAs towards reduced mortality and hospitalization [137]. The OCEAN trial [138] showed that supplemented EPA + DHA in a 2:1 ratio (four capsules of 400/200 EPA/DHA 500 mg per capsule daily) and EPA alone (four capsules of almost pure EPA 500 mg per capsule daily) for 12 weeks led to improved cognitive depressive symptoms related to HF. A clinically relevant improvement in physical function was also reported [138], given that the HF population was composed of 35% patients with HFpEF [90].

Protein supplementation (1.2 g/kg bodyweight per day) associated with low-intensity exercise in 23 obese HFpEF patients for 12 weeks showed benefits on physical and cardiovascular function [139]. In another RCT in a mixed HF population (NCT02240511), branched-chain amino acid (BCAA) supplementation (10 g daily) for 3 months was associated with resistance exercise (RE) [140]. BCAAs are supposed to have an anabolic effect in HF patients, acting as “fuel” during exercise and maintaining muscle mass metabolism [141]. The study did not find benefits from BCAA supplementation and beneficial effects in VO_2_ peak were attributed to resistance exercise program [140].

### Dietary regimens

Dietary regimen studies tested the effectiveness of manipulation of foods and beverages composing the entire diet regimen.

The GOURMET-HF trial [142], including both HFrEF and HFpEF patients, demonstrated that the Dietary Approach to Stop Hypertension (DASH)/sodium-restricted (SDR) diet has a favorable trend in rehospitalization at 30 days. Other studies [143, 144] confirm the effectiveness of the DASH/SDR diet in treating hypertension, reducing 24-h systolic and diastolic BP, and improving diastolic LV relaxation, chamber stiffness, and ventricular-arterial coupling in HFpEF patients. The DASH-DHF 2 trial (NCT01942395) has been designed to confirm the findings of earlier studies in HFpEF patients with history of hypertension. Another clinical trial (NCT05236413) has been recently designed to evaluate the effects of the DASH diet combined with high-intensity interval training (HIIT).

The effects of a low-energy diet (LED) in reduction of myocardial steatosis and improving of diastolic filling in T2D are well known [145]. LED through a low-energy meal replacement plan (MRP) has been proposed as an alternative to achieve weight loss and improve cardiovascular outcomes. This dietary pattern comprises an average of approximately 810 kcal/day (30% protein, 50% carbohydrate, and 20% fat) [146]. Low-energy MRP leads to weight loss, improvement of diabetes-related cardiometabolic risk [147], and reverse of cardiovascular remodeling in obese adults with T2D [146]. The ALLEVIATE trial aims to evaluate the impact of low-energy MRP on symptomatology, physical activity, and QoL in patients with HFpEF and diabetes (NCT04173117). The AMEND trial (NCT05887271) is currently evaluating the results of low-calorie replacement plan in obese HFpEF adults.

The ketogenic diet (KD) is widely adopted to reach weight loss through increased lipolysis [148]. A recent study in a pre-clinical setting [149] showed that ketone supplementation can ameliorate the HFpEF phenotype in mice. Ketone body usage in HFrEF patients showed beneficial hemodynamic effects [150], and clinical studies in HFpEF are awaited. However, caution is needed because of evidenced detrimental effects of KD on cardiovascular health, raising circulating FA levels, which contribute to cardiac lipotoxicity and adversely modifies cardiac muscle energy metabolism [151]. An on-going RCT (NCT04235699) is designed to evaluate the effects of a low carbohydrate KD on exercise tolerance in patients with HFpEF. Another on-going trial (NCT06081543) is designed to evaluate the effects of a low carbohydrate KD versus a low-fat diet on exercise tolerance in participants with HFpEF and diabetes, pre-diabetes, or MetS, or obesity. A prospective pilot study (NCT04942548) aims to examine the impact of low carbohydrate KD on functional and clinical outcomes, and QoL in patients with HFpEF and related pulmonary hypertension HFpEF (PH-HFpEF).

The Mediterranean diet (MedDiet) indicates a dietary pattern including daily consumption of non-refined cereals, olive oil as the principal source of lipids, moderate intake of fish, poultry, potatoes, eggs, and sweets; monthly consumption of red meat, and regular physical activity [152]. The diet involves moderate consumption of alcohol with meals, preferably red wine [152]. Excess alcohol intake might contribute to development of HFpEF and hypertension related organ damage [153, 154]. MedDiet is composed of bioactive molecules, such as ω-3 PUFAs (e.g., EPA, DHA), MUFAs (e.g., oleate), and polyphenols, which confer cardioprotective effects [152]. The MEDIT-AHF trial observed that a greater adherence to MedDiet was associated with a significant reduction in HF hospitalizations following an admission for acute HF, although not with reduced long-term mortality [155]. The PREDIMED trial revealed the positive effects of MedDiet on systemic inflammation markers in patients with HF and MetS [156, 157]. The Hellenic Heart Failure Study, which included 38% of patients with HFpEF, confirmed these positive effects, opening new horizons about its potential benefits [158].

Other dietary regimens might have a positive impact on HFpEF. For instance, plant-based diets such as vegan, lacto-ovo vegetarian, and pesco-vegetarian offer positive effects on cardiometabolic health [159]. Vegetarian diets reduce BP, blood glucose, and lipids levels, with a positive impact on inflammation and body weight [159]. The effects of vegetarian diets in HFpEF should be further explored.

In summary, dietary management for HFpEF exhibits various effects on cardiovascular and metabolic health. Modulation of specific nutrients or manipulation of body composition with CR hold promise but require further validation to pave the way for tailored dietary interventions. Dietary supplementation and regiment studies in HFpEF have, to date, yielded mixed results. While partially dietary supplementation or regiments show promise, more targeted and extensive studies are required to establish their efficacy.

## Physical activity in HFpEF

The American College of Cardiology (ACC)/American Heart Association (AHA) guidelines include a Class 1 recommendation (level of evidence A) for exercise training in patients with HF, without a distinction between HFpEF and HFrEF [160], although the association between physical activity and HFpEF is stronger than with other forms of HF [161]. Evidence suggests amelioration of diastolic function, CRF, exercise capacity, and quality of life (QOL) with exercise training in HFpEF [162–164]. Other studies reported reduction in the hospitalization [165] or fewer cardiac events [166] after exercise interventions in HFpEF. Importantly, a meta-analysis of 6 RCTs [167] reported no exercise-related major adverse events demonstrating the safety of exercise training.

Common indicators of CRF are VO_2_ peak (mL/kg/min) and 6MWD, which represents a valid practical alternative [168]. VO_2_ peak measures the ability to transport (cardiac output) and use (arteriovenous O2 difference) oxygen and is a strong predictor of patients’ functional capacity with significant prognostic value [169, 170]. HFpEF patients present a similar VO_2_ peak to that in age-matched patients with HFrEF, which is severely reduced (by around 30%) when compared with age-matched healthy individuals [171].

### Exercise intolerance and skeletal muscle dysfunction

HFpEF-related-cardiometabolic alterations are linked to worse physical fitness [2]. Patients with HFpEF often exhibit exercise intolerance (EI) and exertional symptoms [172], which are linked to limited O_2_ transport and utilization due to central and peripheral mechanisms [173] and associated with concentric remodeling [174]. Central and peripheral alterations include cardiac (blunted stroke volume augmentation, chronotropic incompetence, exaggerated increase in filling pressures); pulmonary (pulmonary vascular remodeling, impaired gas exchange, pulmonary hypertension); vascular (central artery stiffness, reduced peripheral artery vasodilator response, microvascular dysfunction); and skeletal muscle (reduced mass, excess adipose infiltration, mitochondrial dysfunction) alterations [170]. The latter leads to reduced aerobic exercise capacity of patients with HFpEF, as assessed by 6MWD and VO_2_ peak. Moreover, compromised physical activity and HFpEF-related EI are associated with poor QoL and clinical outcomes and higher incidence of hospitalization [175].

HFpEF-related EI is partly attributed to skeletal muscle dysfunction. Skeletal muscle structure and function in HFpEF are involved in sarcopenic obesity (SO), which is defined as the coexistence of excessive BMI and low muscle mass with multiple comorbidities, excessive visceral adiposity, and heightened systemic inflammation [176]. SO exacerbates cardiometabolic risk, imposing a substantial burden on physical activity and poor QoL [177]. Stratifying HF patients by BMI and body composition could help identify those with SO, where targeted lifestyle interventions to maintain or increase lean mass might be clinically beneficial [178].

HFpEF-related skeletal muscle dysfunction is similar to what is described for HFrEF, and it is not merely a consequence of deconditioning since it develops even when levels of physical activity are maintained during HF development [179, 180]. The pattern of skeletal muscle abnormalities differs from deconditioning, especially as regards fiber-type shift [170]. Abnormal skeletal muscle mitochondrial function [181–183] linked to a perturbed MuSCs homeostasis, involving Hedgehog and apelin pathways signaling has been found [184]. Blunted overload-induced myofiber growth of skeletal muscle is reported in HFpEF despite adequate physical stimulation and ascribed in part to mitochondrial dysfunction [97]. Thus, patients with HFpEF show reduced mitochondrial content and skeletal muscle type I fiber. This contributes to a faster rate of high-energy phosphate depletion during exercise and impaired recovery afterward, as assessed by a study with phosphorous magnetic resonance spectroscopy [181].

Skeletal muscle metabolic abnormalities are linked to functional limitations of patients with HFpEF. On the other hand, evidence suggests that targeting skeletal muscle metabolism might be a promising approach to improving the EI of HFpEF patients [181]. Exercise training leads to peripheral adaptations, such as increased mitochondrial density and function, myoglobin content, capillary density, and blood flow redistribution [185]. Although no significant changes to central artery stiffness are reported, peripheral benefits are observed [94, 163, 186]. Given the high plasticity and predisposition in skeletal muscle [187], increased VO_2_ peak from exercise training results in increased diffusion capacity and oxygen extraction by the exercising muscles [185, 188]. In particular, aerobic training conducted alone or combined with strength training for 3 to 6 months resulted in a safe and effective therapy and enhanced aerobic capacity, endurance, and QoL in HFpEF patients [189].

### Exercise interventions modalities and outcomes

A recent scientific statement from ACC/AHA analyzed data of the 11 latest RCTs on supervised exercise training (SET) for chronic HFpEF subjects [170]. Training approaches range from walking and stationary cycle ergometry to high-intensity interval training (HIIT), strength training, and dancing in both facility setting and home-based training [170]. SET generally occurred 3 sessions per week, from 1 to 8 months, with intensity from 40 to 90% of exercise capacity and individual sessions from 25 to 60 min [170]. SET significantly ameliorates 6MWD and baseline peak VO_2_ by 14%, compared to a reduction in baseline peak VO_2_ by 0.2% in the control group [170]. For comparison, an increase in peak VO_2_ of 6–7% is considered clinically meaningful in patients with HFrEF [190, 191]. However, effects on QoL have been mixed, with some studies concluding no benefits and others demonstrating improved QoL scores [170]. The same applies to cardiovascular and peripheral parameters, showing mixed data among RCTs. Improvements in diastolic function have been demonstrated in some studies, whereas no changes are reported by other investigations [170]. However, the authors conclude that the strength of currently available data on SET and the sparsity of effective therapies for HFpEF provide the rationale for increasing efforts to promote exercise-based therapies for patients [170].

HIIT has recently emerged as an alternative to moderate-intensity continuous training (MICT) in cardiac rehabilitation [192]. HIIT resulted as the best exercise modality in improving V̇O_2_ peak and QoL in a period of about 16 weeks, followed by low intensity training (LIT) with a low-calorie diet as regards effectiveness [193]. HIIT consists of repeated sessions of brief and intermittent exercise that induce ≥ 85% of V̇O_2_ peak, alternated by sessions of rest or LIT for recovery [194, 195]. However, LIT — continuous exercise at a gentle pace, such as walking, light cycling, or slow swimming — with a low-calorie diet resulted as the best lifestyle change in improving 6MWD [193]. Other studies reported the beneficial effects of HIIT in patients with cardiometabolic disorders and chronic diseases, suggesting its effectiveness in improving metabolic health [196–198]. However, the OptimEx-Clin study [199] found no statistically significant differences at 3 months in V̇O_2_ peak by comparing HIIT to moderate intensity continuous training (MICT). Besides this, the findings did not support either HIIT or MICT compared with guideline-based physical activity for patients with HFpEF [199].

In summary, exercise training is highly recommended for HFpEF patients, ameliorating diastolic dysfunction, CRF, exercise capacity, and QoL, while reducing hospitalizations and bringing peripheral beneficial effects, particularly in skeletal muscle. Patients with HFpEF often experience EI due to multiple systemic alterations. In this context, the aerobic capacity and endurance of patients may be enhanced with exercise training, utilizing HIIT as the most effective exercise modality.

## Future perspectives

The relationship between lifestyle interventions and cardiometabolic HFpEF seems to be stronger than in other forms of HF. Given this, dietary interventions can/should be targeted on the metabolic profile of HFpEF patients for precision medicine approaches, to optimize dietary plans considering the unique metabolic disturbances of each patient. For example, as gut microbiome influence in HFpEF is increasingly recognized [200], future dietary interventions may include strategies to modify gut microbiome composition, to enhance its beneficial effects on systemic inflammation and altered metabolism.

The benefits of CR and intermittent fasting in improving metabolic health and inflammation are also emerging. Future studies may focus on optimizing protocols for patients with HFpEF, determining the most effective duration and frequency of fasting periods. Moreover, the nutraceutical properties of some food components reveal potential benefits, targeting specific pathophysiological mechanisms in HFpEF, such as oxidative stress and inflammation. In this regard, further studies on the effect of polyphenols in HFpEF are required, considering their anti-inflammatory and antioxidant properties [201, 202]. Similarly, further investigations should also focus on the effects of spermidine due to its anti-inflammatory and cardioprotective properties.

Combined aerobic and resistance training could provide synergistic effects for patients with HFpEF. Different exercise modalities may be integrated to target both cardiovascular and muscular health, enhancing both cardiometabolic and physical function. HIIT — which emerges as the most effective exercise modality — shows superior benefits in improving exercise capacity and QoL in patients with HFpEF. Future studies may refine HIIT protocols, including intensity and duration to maximize advantages and ensure safety for patients. Importantly, lifestyle intervention studies should consider and further investigate the long-term adherence of patients, which remains a challenge and may attenuate lifestyle intervention benefits.

## Conclusion

Systemic inflammation and metabolic derangements are the main pathophysiological characteristics of HFpEF. Dietary and exercise interventions play a pivotal role in managing both features. Control of body weight, dietary plans, and regular physical activity can significantly improve clinical outcomes in patients with HFpEF. A better understanding of lifestyle intervention modalities will greatly help researchers and clinicians in the management of patients with HFpEF, considering the formulation of multidisciplinary treatment programs. In this perspective, the combination of lifestyle interventions with pharmacological therapies may plausibly show greater effects.
